# Bilateral spontaneous hemotympanum: Case report

**DOI:** 10.1186/1746-160X-2-31

**Published:** 2006-10-04

**Authors:** Dimitrios G Balatsouras, Panayotis Dimitropoulos, Alexandros Fassolis, Georgios Kloutsos, Nicolas C Economou, Stavros Korres, Antonis Kaberos

**Affiliations:** 1 Department of Otolaryngology, Tzanion General Hospital, 1 Afentouli & Zanni, Piraeus, Greece; 2 Department of Otolaryngology, Athens National University, Hippokration Hospital, 114 Vas. Sofias Av., Athens, Greece

## Abstract

**Background:**

The most common causes of hemotympanum are therapeutic nasal packing, epistaxis, blood disorders and blunt trauma to the head. Hemotympanum is characterized as idiopathic, when it is detected in the presence of chronic otitis media. A rare case of spontaneous bilateral hemotympanum in a patient treated with anticoagulants is presented herein.

**Case presentation:**

A 72-year-old male presented with acute deterioration of hearing. In the patient's medical history aortic valve replacement 1 year before presentation was reported. Since then he had been administered regularly coumarinic anticoagulants, with INR levels maintained between 3.4 and 4.0. Otoscopy revealed the presence of bilateral hemotympanum. The audiogram showed symmetrical moderately severe mixed hearing loss bilaterally, with the conductive component predominating.

Tympanograms were flat bilaterally with absent acoustic reflexes. A computerized tomography scan showed the presence of fluid in the mastoid and middle ear bilaterally. Treatment was conservative and consisted of a 10-day course of antibiotics, anticongestants and temporary interruption of the anticoagulant therapy. After 3 weeks, normal tympanic membranes were found and hearing had returned to previous levels.

**Conclusion:**

Anticoagulant intake should be included in the differential diagnosis of hemotympanum, because its detection and appropriate treatment may lead to resolution of the disorder.

## Background

The most common causes of hemotympanum are therapeutic nasal packing, epistaxis, blood disorders and blunt trauma to the head, especially when temporal bone fracture occurs [1,2]. Hemotympanum is characterized as idiopathic, when it is detected in the presence of chronic otitis media [3,4]. In these cases it may be attributed to chronic middle ear effusions, such as granulation and cholesterol tissue originating from a cholesterol granuloma [5].

The aim of this study is to present a rare case of spontaneous bilateral hemotympanum in a patient under medication with anticoagulants.

## Case presentation

A 72-year-old male presented in the emergency ward with acute deterioration of hearing, which occurred during the past 24 hours. In the patient's past medical history aortic valve replacement in May 2004, 1 year before presentation was reported. Since then he had been administered on a regular basis coumarinic anticoagulants. His INR (*International Normalized Ratio*) levels were routinely checked every month and until presentation INR levels between 3.4 and 4.0 were maintained. The latest INR value obtained 2 weeks before the acute deterioration of hearing was 3.6.

**Figure 1:**
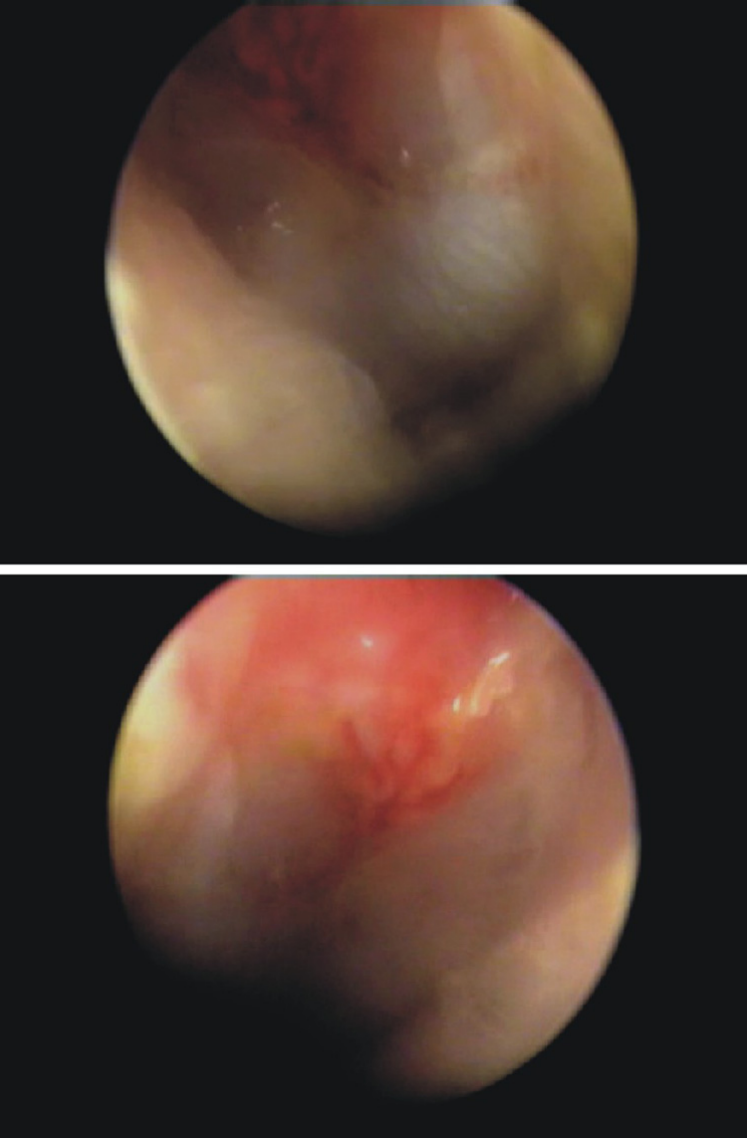
Otoscopic images of bilateral spontaneous hemotympanum, after intake of anticoagulants. Upper: hemotympanum of the left ear; lower: hemotympamum of the right ear.

Otolaryngologic clinical evaluation, including microscopy of the ear, revealed the presence of bilateral hemotympanum (Fig. 1). The tympanic membrane was intact but immobile during the Valsalva maneuver. Other findings were unremarkable and clinical examination proved absence of any other abnormal bleeding on the skin and mucosae. The patient did not mention any abnormal bleeding or bruising in the past. Additionally, the patient's history was clear from chronic middle ear problems or any underlying systemic disorder associated with defective hemostasis and from recent activities related with barotrauma, such as diving, air travel or Valsalva maneuvers. Also, he did not report systemic use of salicylates or any other drugs which could possibly interfere with coagulation, other than the coumarinic anticoagulants. However, the patient mentioned an overdose of nimesulid 2 days ago, for an unrelated reason.

**Figure 2:**
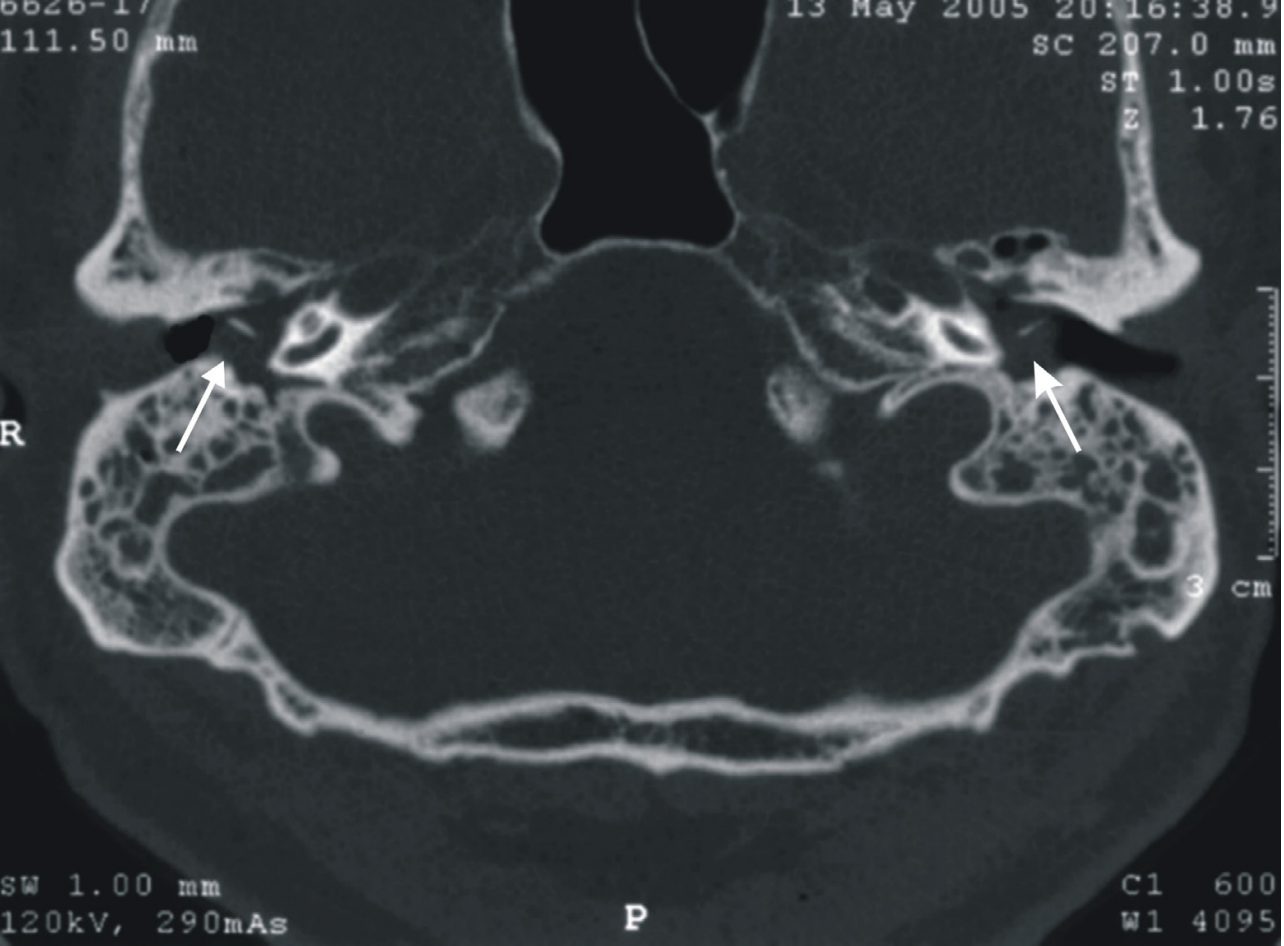
CT scan of the mastoid demonstrating bilateral opacity of the tympanic cavity (white arrows).

An audiogram was obtained, which showed symmetrical moderately severe mixed hearing loss bilaterally, with the conductive component predominating. The air-bone gap for the right ear was 45 dB at 0.25 kHz, 35 dB at 0.5 kHz, 40 dB at 1 kHz, 35 dB at 2 kHz and 25 dB at 4 kHz. The corresponding values for the left ear were 40 dB, 35 dB, 35 dB, 35 dB and 20 dB. Tympanometry revealed flat tympanograms (type B) bilaterally and absence of acoustic reflexes both ipsi- and contralaterally. A computerized tomography (CT) scan was ordered, which showed the presence of fluid in the mastoid and middle ear bilaterally (Fig. 2).

Treatment was conservative. A course of antibiotics taken orally was administered for 10 days, in conjunction with anticongestants. Additionally, we advised temporary interruption of the anticoagulant therapy. The patient had a follow-up otolaryngologic examination after 3 weeks, during which we found that the tympanic membranes were mobile and normal in appearance. Audiometric evaluation showed that hearing had returned to previous levels and acoustic immittance measures were normal. After consultation with the patient's cardiologist, coumarinic anticoagulants were administered again, but in lower doses to maintain INR levels between 2.0 and 3.0. The patient has been followed-up for 6 months at our clinic, and his state is steady, without any new symptoms or signs of bleeding.

Hemotympanum may be easily diagnosed by otoscopy, appearing as partial or total occupancy of the tympanic membrane by bright red, or purple – dark blue colour. The bright red colour of the tympanic membrane is hypothesized to be secondary to oxygen-rich blood owed to recent hemorrhage, whereas the dark colour may originate from oxygen-poor blood from a middle ear effusion or basilar skull fracture [2]. The tympanic cavity derives a rich arterial supply from a number of superficial vessels originating from the external carotid artery [6], which may bleed under certain circumstances.

Hemotympanum may be produced by various causes. Thus, it is important to obtain a detailed medical history from the patient in order to detect the underlying pathology, so that this disorder may be treated properly. The most commonly reported cause of hemotympanum is head trauma [1]. A fracture of the temporal bone, usually resulting from blunt head injury, can produce hemotympanum and hearing loss that can be either conductive, sensorineural or mixed. The conductive element is usually owed to the presence of blood, which may fill the tympanic cavity. Hearing is restored as soon as the blood is absorbed. However, in cases of persistent hearing loss, ossicular disruption may be present.

Occasionally, hemotympanum secondary to therapeutic nasal packing or spontaneous epistaxis was reported [7,8]. Eustachian tube dysfunction is hypothesized to be the cause of this disorder on both occasions, with peritubal lymphatic stasis the most probable pathogenetic mechanism [9]. In cases of epistaxis without nasal packing, retrograde reflux of blood through a patulous Eustachian tube may be implicated [8].

Several other rare causes of hemotympanum should be also considered. In advanced tuberculosis blood may penetrate into the middle ear during haemoptysis [1]. Barotrauma of the middle ear is another possible cause of hemotympanum. The most common causes of barotrauma today are from the use of the Self-Contained Underwater Breathing Apparatus (SCUBA), air travel, and from hyperbaric oxygen chambers. Edmonds from the Australian Diving Medical Center [10], devised a grading system for middle ear barotrauma, based on the otoscopic appearance of the tympanic membrane. The grading scale is from Grade 0, when a patient experiences symptoms of middle ear barotrauma but no physical findings are present, to Grade V, when a perforation of the tympanic membrane is apparent. Hemotympanum, evidenced by blueness and bulging of the tympanic membrane, represents Grade IV in this scale.

In patients who are on anticoagulants or suffer from leukaemia, spontaneous bleeding within the temporal bone may also occur [11]. In our patient regular uptake of anticoagulants was most probably the cause of spontaneous bilateral hemotympanum. To our knowledge such a case has never been reported in the literature so far. Uptake of nimesulide might be an aggravating factor for the clinical manifestation of this condition. Antiinflammatory drugs are known to interfere with blood coagulation. Although evidence is still inconclusive, it appears that nimesulide may inhibit platelet aggregation and, additionally, may inhibit thromboxane A2 formation by platelets at low concentration [12]. It has been reported that although nimesulide does not usually affect the response to coumarin anticoagulants, a few patients may show some increase in anticoagulant effect [13].

In all cases, differential diagnosis between secondary and idiopathic hemotympanum should be made. The latter is associated with the presence of cholesterol granuloma derived from granulomatosus mastoiditis and is characterized by purple colour of the tympanic membrane [5]. Cholesterol granuloma can be diagnosed by MRI, as it has a characteristic appearance in T1- and T2- weighed images. Main et al. [14] have produced successfully cholesterol granuloma in monkeys under persistent obstruction of the Eustachian tube.

Treatment of hemotympanum should be, initially, conservative. Myringotomy and insertion of a ventilation tube is indicated for treatment when the condition persists after one month [1]. In these cases, the presence of a glomus tumor, either involving the jugular bulb (glomus jugulare) or confined to the middle ear or mastoid (glomus tympanicum), should be considered [15]. Clinical detection of this disorder is often difficult, and usually, contrast-enhanced CT scans in conjunction with angiography are needed.

In our patient treatment was conservative. Additionally, we ordered termination of the anticoagulant administration for a short period of time and prescribed a course of antibiotics and nasal anticongestants until the absorption of blood. Resolution was observed after 3 weeks, so that adjunctive therapeutic measures were not necessary.

## Conclusion

Detailed patient's history may lead to accurate diagnosis of the cause of hemotympanum. We should always search for rare causes of hemotympanum, such as intake of anticoagulants, because appropriate treatment may result in resolution of the disorder.

## Competing interests

The author(s) declare that they have no competing interests.

## Authors' contributions

DGB diagnosed the case and drafted the manuscript. PD diagnosed the case and assisted in drafting the manuscript; AF assisted in the diagnostic laboratory work-up of the patient; GK assisted in the diagnostic laboratory work-up of the patient; NCE participated in the design and coordination of the study and assisted in preparing the manuscript. SK examined the patient and assisted in drafting the manuscript. AK has been involved in revising the manuscript critically for important intellectual content.

All authors read and approved the final manuscript.
